# Two-Person Approaches to Studying Social Interaction in Psychiatry: Uses and Clinical Relevance

**DOI:** 10.3389/fpsyt.2020.00301

**Published:** 2020-04-24

**Authors:** Yafeng Pan, Xiaojun Cheng

**Affiliations:** ^1^ School of Psychology, Shenzhen University, Shenzhen, China; ^2^ Department of Clinical Neuroscience, Karolinska Institutet, Stockholm, Sweden

**Keywords:** two-person approach, social interaction, psychiatry, application, intervention

## Abstract

Social interaction is ubiquitous in human society. The two-person approach—a new, powerful tool to study information exchange and social behaviors—aims to characterize the behavioral dynamics and neural mechanisms of real-time social interactions. In this review, we discuss the benefits of two-person approaches compared to those for conventional, single-person approaches. We describe measures and paradigms that model social interaction in three dimensions (3-D), including eye-to-eye, body-to-body, and brain-to-brain relationships. We then discuss how these two-person measures and paradigms are used in psychiatric conditions (e.g., autism, mood disorders, schizophrenia, borderline personality disorder, and psychotherapy). Furthermore, the advantages of a two-person approach (e.g., dual brain stimulation, multi-person neurofeedback) in clinical interventions are described. Finally, we discuss the methodological and translational challenges surrounding the application of two-person approaches in psychiatry, as well as prospects for future two-/multi-person studies. We conclude that two-person approaches serve as useful additions to the range of behavioral and neuroscientific methods available to assess social interaction in psychiatric settings, for both diagnostic techniques and complementary interventions.

## Introduction

The scientific study of the neurophysiological mechanisms that underlie social processes is undergoing a major paradigm shift: moving from the examination of single brains to the simultaneous acquisition of data from multiple brains and their interaction [see, e.g., our recent contributions: (1–6); see also (7–9) for reviews]. Within this fast-emerging area of research, we focused on recent advances that examined the relationship between eye gaze/body movement/brain activity recorded from interacting dyads under psychiatric settings. Thus, we attempted to characterize *social interaction in psychiatry* within a two-person framework (9–11).

In the present review, we will first briefly introduce the two-person approach and its benefits compared to those of a single-person approach, as well as potential interpersonal paradigms/markers derived from this approach. We will then focus on two-person studies in two aspects: first, the applications of the two-person approach in multiple cases of psychiatric conditions (e.g., autism, mood disorders, schizophrenia, borderline personality disorder, and psychotherapy), and second, the potential benefits of the two-person approach in psychiatric interventions (e.g., behavioral intervention, dual brain stimulation, multi-person neurofeedback). Finally, we will discuss challenges and future prospects of the applications of the two-person approach.

### What Is the Two-Person Approach?

Human society is organized socially. Despite the interactive nature of human social behaviors, conventional neuroscientific studies investigating social cognitive processes have typically been restricted to isolated individual behaviors, leaving the dynamic (neural) interactions between individuals incompletely understood. Methodological advances allow researchers to address this issue by developing a novel technique termed “hyperscanning” or “hypermethod” [e.g., using electroencephalography (EEG) (12), functional near-infrared spectroscopy (fNIRS) (13), or functional magnetic resonance imaging (fMRI) (14)]. This technique first highlights the simultaneous consideration of two individuals in an interacting dyad.

In 2013, Schilbach and colleagues further advanced this field and formally proposed the theoretical framework of “second-person neuroscience” (11) or “two-person neuroscience” (15). Accordingly, recent years have seen fruitful empirical evidence of this two-person approach [for a review, see (9)]. The two-person approach has a basic assumption: behavioral and neural mechanisms supporting social cognition within the context of a real-time reciprocal social interaction are distinguishable from those within the context of social observation (without interaction). To further define two-person studies properly, two criteria were proposed: (*i*) social interactions should occur in real time and be reciprocal, and (*ii*) social interactions elicit psychological engagement (feeling of involvement with one another) between interacting partners. Studies having one of the two criteria could be seen as two-person studies.

Note that *two*-person approaches do not necessarily mean that investigations should be conducted only with *two* interacting individuals; one can also develop variants by monitoring *multiple* persons because social interaction could also take place in multi-person situations (16).

### Is “Two” Indeed Better Than “One”?

As described in the previous section, human social behaviors have an interactive nature. To characterize the dynamic social interaction between individuals, it is imperative to adopt the two-person approach. However, one important question should be addressed first:*is “two” indeed better than “one”*? Several neuroimaging studies have attempted to address this issue (6, 17–19). Using fNIRS-based hyperscanning and machine learning approaches, Pan et al. found that two-brain measures served as a better neural-classification feature than single-brain measures (6). Specifically, machine learning techniques were reported to be more successful when decoding instructional approaches from instructor-learner brain coupling data than when using a single-brain method. Supporting these findings, previous fMRI studies reported that two-brain measures, such as brain-to-brain similarities, are more sensitive and better suited to track inter-personal influences, such as social network proximity (17); friends showed more similar neural responses to naturalistic movies, and such similar neural responses decreased with increasing social distance between friends. Monitoring and measuring two individuals simultaneously uncovered additional information beyond conventional single-brain approaches (18, 19). For example, compared to the single-brain method, which reflects a mixture of both neuronal components (i.e., stimulus-induced neural processes) and non-neuronal components (e.g., intrinsic neural processes and non-neuronal noise), two-brain measures using fMRI isolated stimulus-related inter-brain correlations (18). We believe that these prior studies are sufficient to imply that “two” performs better than “one” in several aspects during real-time social interaction; however, more research is needed to clarify the assets of the two-person approach compared to those of the single-person approach.

It is important to note that we do not claim that adopting the single-person approach to investigate the social cognitive process is useless – this contention would discredit various classic and ongoing investigations in this field [e.g., (20–22)]. Instead, we propose that using the two-person approach would add additional value to the exploration of dynamic and truly social interaction, thus advancing our understanding of both behavioral and neural mechanisms underlying human social behaviors.

### Modeling Social Interaction in 3-D

To take advantage of the two-person approach, the field calls for interactive paradigms and interpersonal markers of real-time social interaction (23, 24). Here, we highlight three dimensions (3-D) that characterize the behavioral and neural mechanisms of social interaction. These paradigms/markers allow us to model real-time social interaction in 3-D: eye-to-eye, body-to-body, and brain-to-brain.

#### Eye-to-Eye

Gaze behavior is critical in social interaction and in communication in particular. While many studies have investigated the role of gaze behavior in social observation, research about a person's interactive gaze allowing eye-to-eye contact and face-to-face interaction is still lacking. Pfeiffer et al. reviewed novel approaches to investigate the neural systems that support social gaze behavior, thus requiring active social engagement (25); these novel approaches include interactions with virtual agents (26), live interactions *via* videos (27), and dual eye-tracking setups (28).

Regarding dual-eye-tracking setups, in the last decade, we have seen fruitful applications of the eye-to-eye paradigm in investigating neural mechanisms of social interaction. For example, Saito and colleagues initiated a combination of fMRI hyperscanning and dual eye-tracking. With this novel setup, they found that paired subjects showed higher inter-individual neural synchronization in the right inferior frontal gyrus *during* mutual gaze and joint attention activities than non-paired subjects (28). Using EEG hyperscanning in an eye-to-eye (face-to-face) situation, Lachat et al. found that the joint attention condition—compared to the no-joint attention condition—induced an 11-13 Hz power decrease over left centro-parieto-occipital regions (29). As Lachat et al. suggested, the power decrease might reflect attention mirroring, social coordination, and mutual attentiveness associated with joint attention. Hirsch et al. combined a two-person eye-tracking system and fNIRS recordings. The results revealed that fronto-temporo-parietal neural systems synchronize within and across brains during live eye-to-eye contact, in contrast with the results for an eye-to-picture gaze (30).

#### Body-to-Body

Body movement is an important nonverbal cue and signal for social interaction. The coupling between hand/body movements acts as an index for implicit social interaction (31). For example, Yun et al. found that synchrony of both fingertip movement and neural activity between two individuals in a dyad increased after a cooperation interaction. In more complex social interactions, nonverbal interpersonal coordination (body sway) among people was indicative of leadership in joint music making (32).

Furthermore, recent advances also reported that body-to-body coupling was associated with many positive outcomes, including prosociality (2), therapeutic alliances (33), mentalizing (34), and closeness (35). Using a combination of a two-person paradigm and fNIRS recordings, Hu and colleagues found that the manipulation of body-to-body synchrony predicted subsequent prosocial behaviors. Brain-to-brain synchronization between the two participants during ongoing movements might be a potential underlying mechanism. At the behavioral level, Ramseyer et al. (33) quantified nonverbal synchrony between the patient's and therapist's movements. They found that nonverbal synchrony reflected the relationship quality; synchrony was associated with symptom reduction. Baimel et al. found that behavioral synchrony between partners fostered mentalizing capacities. Synchrony increased the mental state attribution to interacting participant dyads (34). Another behavioral study explored the influence of motor synchrony on the experience of intimacy (35). Specifically, the authors examined whether body-to-body synchrony between partners instilled a sense of intimacy. The results suggested that synchrony was strongly associated with intimacy and possibly promoted closeness in intimate situations.

Another subdomain of the body-to-body relationship concerns peripheral physiological signals, including the heart rate, electrodermal activity, and respiration. The relationship between the physiological activity of two or more people is referred to as “interpersonal autonomic physiology” or “physiological synchrony” (36). The concept of physiological synchrony has been incorporated into a wide range of contexts to investigate its relation with a number of social behaviors, including cooperation (37), singing (38), and romantic interaction (39). Specifically, in the field of psychiatry, physiological synchrony serves as a useful tool to track psychotherapy processes (40–42). In 2016, Koole and Tschacher reviewed clinical studies on therapeutic alliances and interpersonal synchrony and then integrated both concepts into the interpersonal synchrony model of psychotherapy. In a later empirical study, Tschacher and Meier explored physiological synchrony in naturalistic psychotherapy sessions and found that synchrony correlated with the therapeutic alliance and psychotherapy session reports.

#### Brain-to-Brain

The most recent neuroimaging work in this field shifted the focus on single-brain functioning toward two-brain communication during real-time social interaction. As mentioned above, this shift is boosted by a fast-developing technique: “hyperscanning”. The concept of hyperscanning was first proposed by Montague et al. (14). In their commentary paper, the authors described simultaneous neuroimaging during linked social interactions; specifically, participants could interact with each other while their brain activity was simultaneously recorded. Regarding data analysis, hyperscanning setups enable us to effectively quantify the relationship between two brain activities. A common finding derived from previous hyperscanning studies is that brain activity from two interacting participants in a dyad tends to be “coupled together”, creating a joint networked state. This phenomenon is usually called “brain-to-brain synchrony/synchronization” (16) or “interpersonal brain/neural synchronization” (4, 5, 43–45).

The mechanisms of brain-to-brain synchrony are still debated. Although some researchers see brain-to-brain synchrony, *per se*, as a mechanism for social behaviors [e.g., (8)], others claim that synchrony is not a mechanism in itself but a measurable reflection of the underlying neural computations that support some psychological processes [e.g., (16)]. Dikker and colleagues proposed “shared attention” as a possible source of brain-to-brain synchrony by successfully demonstrating a positive relationship between alpha band power (a well-characterized index of attention) and synchrony measures. This account is also in line with a series of past studies using mutual gaze tasks (28, 46–48). Apart from this account of shared attention, other studies also posited that social signals (such as gaze, gestures, or vocalizations) could promote mutual temporal alignment of the brains involved, leading to a joint networked state to facilitate information transfer (5, 49).

Note that this review is not intended to be a comprehensive review of two-person approaches, given the excellent reviews previously published on this topic (9, 11, 50). For this reason, we did not go into depth about any techniques but instead refer readers to **Table 1** that briefly describes the commonly used measures and other empirical work [e.g., (58–60)]. Additionally, compared to emerging two-person neuroscience endeavors (i.e., brain-to-brain), two-person behaviors (i.e., eye-to-eye and body-to-body) have long been monitored to investigate social interaction. With this in mind, we mainly discussed behavioral paradigms that were (potentially) related to two-person neuroscience, as that is where our novelty lies.

**Table 1:** Several commonly used measures in two- or multi-person studies.

Measure	Description	Example References
Motion energy analysis	Computing pixel changes across video frames and generate motion energy time series for both participants during interaction	(33, 51)
Windowed cross-correlation	Tracking the movements of two variables or sets of data relative to each other	(33, 51, 52)
Phase locking value	Measuring the consistency of the phase-difference	(3, 53, 54)
Circular correlation coefficient	Measuring the circular covariance of differences between the observed phase and the expected phase	(55–57)
Wavelet transform coherence	Measuring local correlation between two signals as a function of both frequency and time	(1, 4–6)
Granger causality analysis	Estimating the directional coupling	(1, 4, 5, 45)

Readers should keep in mind that the three aforementioned dimensions (eye-to-eye, body-to-body, and brain-to-brain) are not isolated but are strongly associated with each other. For instance, eye contact synchronization might occur in parallel with brain activity synchronization (28, 30), and body-to-body coupling could be associated with brain-to-brain coupling (2, 31). These novel approaches facilitate the investigation of behavioral dynamics and neural mechanisms of social behaviors (50) and thus yield new insights into the field of *social interaction in psychiatry*.

## Recent Applications of the Two-Person Approach in Social Interactions Under Psychiatric Settings

### Autism

Autism spectrum disorder (ASD) is a neurodevelopmental disorder characterized by social deficits in communication and inter-personal interactions, as well as non-social deficits in repetitive behavior (61). Social deficits in communication in individuals with ASD are reflected in various aspects, such as joint attention (62–64), motor imitation (65), and interpersonal coordination (66, 67).

Joint attention is a set of nonverbal behaviors, including eye gaze, pointing, and showing, which are used to reference outside objects during a communicative exchange (62). Generally, joint attention occurs within the context of a social interaction, when one person directs another person's attention to an object (*initiating joint attention*), and the second person's attention follows (*responding to joint attention*). Previous studies have established impaired joint attention in children with ASD (62) and that the early development of joint attention predicts future language and social cognitive skills in children with ASD (68, 69); therefore, joint attention skills are critical targets of intervention for this population.

Atypical joint attention behaviors and brain activation patterns have been observed in individuals with ASD when they were required to view images or movies of real or virtual people (63, 70). However, these traditional experimental setups (i.e., viewing images or movies) might not be a promising method to disclose the neural basis of *initiating joint attention* in individuals with ASD. Concerning this issue, Redcay et al. adopted a dual-video setup that allowed for a face-to-face interaction between the subject and experimenter *via* video during fMRI data collection. By using the two-person approach, they depicted activation patterns related to *initiating joint attention* and *responding to joint attention* in both the ASD group and the normal control group (27). Compared with the normal group, the ASD group showed a reduced brain activation difference between joint attention conditions (including *initiating joint attention* and *responding to joint attention*) and solo attention conditions in the dorsal medial prefrontal cortex and right posterior superior temporal sulcus. Distinct regions included the ventromedial prefrontal cortex for *responding to joint attention* and the intraparietal sulcus and middle frontal gyrus for *initiating joint attention* (27). The lack of differentiation was further characterized by reduced activation during joint attention conditions and relative hyperactivation during solo attention conditions (71).

Although Redcay and colleagues applied the two-person approach and adopted a live face-to-face communication task in their study, they used a single fMRI setting and measured a single brain in an isolated manner. To elucidate the neural substrates of direct, real-time interactions between ASD patients and normal subjects, Tanabe et al. (72) conducted the first fMRI-based hyperscanning study in ASD individuals by using the mutual gaze paradigm developed by Saito et al.. They found that compared to the normal–normal pairs, ASD–normal pairs exhibited less accurate gaze direction detection and less prominent inter-brain coherence in the right inferior frontal gyrus during eye contact (72). The findings suggest that the impairment of joint attention in ASD could be related to the difficulty in understanding shared intention through eye contact, which is represented by reduced inter-subject synchronization of cortical regions including the right inferior frontal gyrus.

Apart from impaired joint attention, most individuals with ASD have deficits in interpersonal motor imitation and coordination (65–67). A related study has shown that in individuals with ASD, a higher degree of autistic traits (i.e., higher Autism Spectrum Quotient score) could predict a lower ability to modulate movements to coordinate with normal individuals (i.e., social interactive tasks) but not differences in movement preparation and planning with a non-biological stimulus (i.e., non-social tasks) (66). This finding suggests that the failure of individuals with ASD to coordinate with others was not due to basic motor or executive function difficulties. Furthermore, the performance of individuals with ASD regarding interpersonal motor coordination could possibly depend on the social skill ability of the individuals with whom they are paired (73). Pairs of participants with widely differing Autism Spectrum Quotient scores performed better than pairs with similar Autism Spectrum Quotient scores in the inter-personal rhythmic movement task. Specifically, participants with relatively higher Autism Spectrum Quotient scores tended to precede their partners in the task.

Recently, Wang and colleagues conducted fNIRS-based hyperscanning studies to further explore brain-to-brain coupling during interpersonal coordination tasks between children with ASD and normal partners (i.e., parents in the study). The results showed that compared to solo and non-interactive behaviors, coordinating interactions with their parents could elicit increased inter-personal neural synchronization in the frontal cortex of children with ASD (67). Neural synchronization was further found to be modulated by the children's autism symptoms and covaried with their cooperation task performance. That is, children with severe autism symptoms showed worse behavioral performance and less neural synchronization with their parents during coordination than children with less severe symptoms.

### Mood Disorders

Patients suffering from mood disorders, e.g., major depressive disorder and bipolar disorder, showed atypical interpersonal communication according to their mood state (61). Previous studies have employed fMRI to explore cognitive and emotional dysfunctions and found altered activation of the amygdala, as well as the frontal, cingulate, and temporal cortices, in patients with major depressive disorder and bipolar disorder during various cognitive tasks (74). However, brain activation during conversation has not yet been investigated in patients with mood disorders due to methodological difficulties.

In 2014, Takei and colleagues conducted a fNIRS study in which major depressive disorder and bipolar disorder patients performed a face-to-face conversation with an interviewer who was selected from the hospital staff and had not been previously acquainted with the participants (i.e., a two-person situation with a focus on the patient's brain). In their study, patients' frontal and temporal lobe activation levels were measured during the conversation condition (including speech and listening phases) and control condition (including syllables and silent phases). The results showed less activation in the left dorsolateral prefrontal and left frontopolar cortices in major depressive disorder and bipolar disorder patients than in normal individuals, as well as a rapid decrease in bilateral frontopolar activation in major depressive disorder and bipolar disorder patients. Particularly, in patients with major depressive disorder, the average amount of signal change over time in the frontopolar cortex was positively correlated with their Global Assessment of Functioning scores; in patients with bipolar disorder, the average brain activation during conversation was negatively correlated with the age of onset in the right dorsolateral prefrontal cortex and both middle temporal lobes (75). These findings suggest that both continuous activation and rapid change may reflect the pathophysiological characteristics of major depressive disorder and bipolar disorder.

### Schizophrenia

Schizophrenia is marked by poor social-role performance and social-functioning deficits that are well reflected in interpersonal communication (61). Such social deficits could be captured by body-to-body dynamics. For example, Kupper and colleagues found that a low level of nonverbal synchrony was associated with negative symptoms, low social competence, impaired social functioning, and low self-reported competence. Negative symptoms were more prominent when patients reduced their imitation of the movements of the interactant; in turn, positive symptoms were more prominent when interactants reduced their imitation of patients' movements (51).

The social deficits shown by patients with schizophrenia could be associated with reduced volume and/or reduced gray matter activation in specific brain regions, such as the temporal lobe, ventromedial prefrontal cortex, and cingulate cortex (76, 77). Takei et al. used fNIRS to investigate frontal and temporal lobe activation in patients with schizophrenia during the conversation (i.e., a two-person situation with a focus on the patient's brain). The results showed that patients with schizophrenia, compared to normal controls, were characterized by decreased activation in the bilateral temporal lobes and right inferior frontal gyrus during the conversation task (78). The decreased activation in the related brain regions negatively correlated with disorganization and negative symptoms suggested that the disorganization and negative symptoms observed in patients with schizophrenia in clinical situations are related to dysfunction of the left temporal lobe and right inferior frontal gyrus. In addition, frontal lobe dysfunction was also reported to be linked to difficulties in gesture planning and execution (79), which might explain the poor social functioning in schizophrenia patients.

### Borderline Personality Disorder

Borderline personality disorder is characterized by repeated interpersonal conflict and unstable relationships (61). Bilek and colleagues recently explored the neurobiological mechanism of social interactive deficits in borderline personality disorder. In their study, current borderline personality disorder patients and remitted borderline personality disorder patients were recruited to perform a joint attention task with normal participants. Compared with the normal-normal pairs, normal-current borderline personality disorder pairs showed reduced interpersonal brain connectivity. Remarkably, for remitted patients, interpersonal brain connectivity was restored. These findings emerged only in the study of information flow between dyads and were not associated with any between-group differences in individual brain structure or function, indicating the necessity of two-person approaches. Cross-brain measurements, therefore, deliver state-associated biomarkers that may help to guide diagnostic and therapeutic procedures in the future (80).

### Psychotherapy

As described above, individuals with psychiatric disorders have social deficits that reflect verbal or nonverbal coordination with others to some extent. It is worth noting that the synchrony, especially nonverbal synchrony, between patients and therapists is also highlighted during psychotherapy.

Ramseyer et al. (33) found higher nonverbal coordination in genuine interactions (i.e., real pairs of patients and therapists) in contrast with pseudo-interactions (i.e., random pairs of patients and therapists). More importantly, nonverbal coordination was associated with patients experiencing high quality relationships and high self-efficacy (33). Other studies showed that nonverbal synchrony between patients and therapists could be modulated by therapeutic approaches (81) and varied by disorder (82).

Recently, researchers attempted to disclose the neural mechanisms underlying behavioral synchrony during psychotherapy. In a preliminary study, typical students were recruited as clients, and they were required to have interactions with professional counsellors. By using the fNIRS-based hyperscanning technique, researchers recorded the brain activation of both clients and counsellors in the frontal cortex and right temporoparietal junction during the psychological counselling phase and the chatting phase (83). Better working alliances and increased interpersonal brain synchrony in the right temporoparietal junction between clients and counselors were observed during psychological counseling (versus chatting). Such inter-personal brain synchrony was correlated with the bond of the working alliance. This study refines the neural explanation of behavioral synchrony during psychotherapy.

Briefly, interpersonal body and brain synchrony could play important roles in the processes of psychotherapy. The lack of coordination during psychotherapy may be a risk factor for the condition's recurrence (84). The findings provide insights for psychological interventions for psychiatric disorders.

## Interpersonal Body and Brain Coupling Offer Insights for Psychological Interventions for Psychiatric Disorders

### Manipulation of Inter-personal Body Synchrony

Given that synchrony has also been associated with the outcome of psychotherapy, “moving together” could possibly be an efficient means to improve psychiatric patients' social dysfunction. Notably, the idea of using imitation and synchronization in clinical interventions to target social functions has a long tradition in dance/movement therapy in general and in working with children with ASD in particular (85–87). Some studies have provided empirical evidence for this notion. For example, a seven-week intervention study focusing on movement mirroring showed that young adults with ASD reported improved well-being, body awareness, self–other distinction, and social skills after the intervention (88). Moreover, patients treated with an interpersonal movement imitation and synchronization intervention showed a significantly larger improvement in emotional inference than those treated with a control movement intervention that focused on individual motor coordination (89).

Interventions targeting social synchronous behavior on social functions can even positively affect two-year-old toddlers with ASD (90). In the study by Landa et al., toddlers with ASD were randomized to either a classroom-based inter-personal synchrony intervention (including imitation, joint attention, and affect sharing) or a non-inter-personal synchrony intervention. It was found that after approximately 200 hours of interpersonal synchrony interventions (versus non-interpersonal synchrony interventions), toddlers showed enhanced socially engaged imitations paired with eye contact with the examiner and demonstrated a trend toward higher levels of nonverbal cognition during posttest assessments (90). The study provided evidence for plasticity in these developmental systems in toddlers with ASD.

Notably, the interventions could initially be conducted *via* a computer-mediated interference. It was reported that individuals with high-functioning autism showed a reduced sensitivity to the other person's responsiveness to one's own behavior when they were required to have real-time sensorimotor interaction with normal individuals; however, they performed equally well as controls under the highly simplified, computer-mediated, embodied form of social interaction. This finding supports the increasing use of virtual reality interfaces to help people with ASD better compensate for their social disabilities (91).

### Manipulation of Inter-personal Brain Synchrony

Related studies have revealed that interpersonal brain synchrony reflects social dysfunctions and intervention effects to some extent (80, 83). Thus, it could be possible to improve the social communication and interpersonal relationships of patients with psychiatric disorders by manipulating interpersonal brain synchrony.

To date, some studies have applied transcranial alternating current stimulation (tACS) to specific brain regions of interacting persons to directly examine the relationship between interpersonal brain synchrony and behavioral synchrony (92, 93). For example, Novembre and colleagues induced beta band (20 Hz) oscillations over the left motor cortex in pairs of individuals who both performed a finger-tapping task with the right hand and found that in-phase 20 Hz stimulation enhanced inter-personal movement synchrony compared with anti-phase or sham stimulation, particularly for the initial taps following the preparatory period. However, in the study of Szymanski et al., both the same-phase-same-frequency and the different-phase-different-frequency conditions were associated with greater dyadic drumming asynchrony relative to the sham condition. The inconsistent findings might be related to the different stimulation protocols and experimental paradigms, which need to be verified.

Apart from dual-brain stimulation, neurofeedback is also a promising approach to manipulating brain activity. There is growing evidence to support the idea that a single participant's brain activity can be self-regulated with neurofeedback, yielding specific behavioral effects [see (94, 95) for a review]. During the past several decades, the technique of neurofeedback has been applied to patients with psychiatric disorders (e.g., major depressive disorder, personality disorder, and schizophrenia) to relieve psychiatric symptoms [see (96) for a review]. Previous studies have revealed that a neurofeedback tool that tracks human interaction at the neural level has potential clinical applications for the diagnosis and treatment of social cognition disorders. For example, persons with autism may respond better to explicit cues *via* technological interfaces than to human cues (91).

## Challenges

### Artifacts and Sample Size

To study the behavioral and neurophysiological mechanisms underlying social interactions, two-person approaches call for highly ecologically valid experimental paradigms. At the methodological level, compared to fMRI and EEG data, fNIRS data offer the advantage of capturing brain activity in realistic social situations while being less affected by motor artifacts (97). Some video-based techniques or peripheral devices restrain participants even less (e.g., motion energy analysis, actigraphy).

However, high ecological validity comes at the cost of having unavoidable artifacts. The (neural) signal is potentially contaminated by at least two factors. First, motion artifacts are likely to be generated in unconstrained environments (98, 99), affecting the reliability of data. Second, spontaneous systemic effects from neural activity or peripheral physiological fluctuations may affect signal quality (100). These artifacts could be mitigated by several artifact correction methods. However, good *post hoc* data processing is never better than good data collection. Future studies should simultaneously consider the ecological validity and motion/systemic artifacts when applying two-person approaches.

A common issue in two-person neuroscientific research relates to the sample size. “Two-person” is a fancy technique, but it also means that a larger sample size would be required. For example, 60 participants indicate a sample size of 60 for single-person studies but only half for two-person studies in many cases (i.e., 60 participants would lead to a sample size of 30 for two-person studies; and an even smaller sample size for multi-person studies). One is encouraged to define the sample size prior to formal experiments by conducting statistical power analyses, e.g., using the G*power toolbox (101). Future studies are needed to consolidate the previous findings from two-person studies by enlarging the sample size to increase statistical power.

### Statistical Methods of Assessing Synchrony

There are several techniques that estimate the covariance or directional coupling of time series generated from two interacting partners in previous studies. These include the phase locking value [(53); see also (3, 54)], wavelet transform coherence [(102); see also (4, 13)], windowed cross-correlation [(52); see also (33, 51)], and Granger causality analysis [(103); see also (5, 32, 45)].

However, when evaluating data analyses for two-person data, there is currently no uniform analytical pipeline (i.e., statistical methods for assessing synchrony). Different two-person studies adopt distinct analysis strategies (e.g., with vs. without filtering), making the findings less congruent. In an endeavor to achieve transparency, consistency, and repeatability, future research should reach a consensus on common analysis guidelines. This practice will largely facilitate the replication of findings and their interpretations. Recent advances have seen some efforts in this direction [e.g., (104)].

Indeed, different methods aim at addressing distinctive research questions. For example, interpersonal *phase* synchronization between two participants in a dyad could be addressed using phase locking value or wavelet transform coherence methods, whereas the former was commonly used in behavioral and EEG studies and the latter was widely used in fNIRS research. Windowed cross-correlation not only provides information regarding simultaneous synchrony but can also reveal lagged information (i.e., which time series is leading the other). Granger causality also provides suggestions for coupling directionality, in case one is interested in exploring the direction of information flow between individuals. Researchers should utilize suitable statistical methods for assessing synchrony, targeting specific research aims.

### Clinical Translation Application

Current two-person studies are still facing technical and methodological challenges, making the findings difficult to interpret and controversial for direct clinical translation. In addition, considering the limited sample size in previous two-person studies, it is rather inappropriate to generalize the laboratory findings to real-life psychiatric applications. Notwithstanding, previous studies pave the way for the use of two-person approaches in practical psychiatric settings. For example, it was reported that body-to-body synchrony reflected relationship quality and outcomes in psychotherapy (33). This implies that interpersonal markers could be potential tools to aid diagnostic procedures. Additionally, dual brain stimulation was reported to foster behavioral coordination and improve social interactions (92, 93). This is relevant for complementary treatments for social disorders, such as autism.

## Conclusions and Future Perspectives

In sum, two-person approaches are promising tools for studying social interaction, particularly in the field of psychiatry. Although still facing several methodological and translational challenges, the two-person approach has its benefits compared to the conventional single-person approach when studying dynamic social interactions. The use of two-person approaches in psychiatry facilitates advancements in our understanding of the mechanism of atypical social interaction in the fields of autism, mood disorders, schizophrenia, borderline personality disorder, and psychotherapy. Two-person approaches also show promise in clinical interventions when combined with brain stimulation and neurofeedback techniques.

A future direction is to integrate the two-person approaches with computational modeling techniques (105), which may help further empower a better understanding of the computational mechanism of social interaction in psychiatry. Another direction is to manipulate the situations during social interaction using virtual reality (106) and test the effects of social factors, such as interpersonal distances and angles on eye-to-eye, body-to-body, and brain-to-brain communications. Eventually, as psychiatric disorders are strongly influenced by genetic factors (107), future studies are also encouraged to combine two-person approaches and multivariate genetic models.

## Author Contributions

YP and XC wrote the manuscript.

## Funding

Support was provided by the National Natural Science Foundation of China (31800951), the Natural Science Foundation of Guangdong (2018A030310431), the Foundation for Distinguished Young Talents in Higher Education of Guangdong (2017KQNCX172), and the Natural Science Foundation of SZU (2018053).

## Conflict of Interest

The authors declare that the research was conducted in the absence of any commercial or financial relationships that could be construed as a potential conflict of interest.
